# Effects of a 12-Week Digital Care Program for Chronic Knee Pain on Pain, Mobility, and Surgery Risk: Randomized Controlled Trial

**DOI:** 10.2196/jmir.9667

**Published:** 2018-04-25

**Authors:** Gabriel Mecklenburg, Peter Smittenaar, Jennifer C Erhart-Hledik, Daniel A Perez, Simon Hunter

**Affiliations:** ^1^ Hinge Health, Inc San Francisco, CA United States; ^2^ Department of Orthopaedic Surgery Stanford University Medical Center Stanford University Stanford, CA United States

**Keywords:** osteoarthritis, knee, chronic pain, exercise, education, cognitive behavioral therapy, computers, handheld, coaching, non-invasive, digital health, digital therapy, digital care program

## Abstract

**Background:**

Chronic knee pain, most commonly caused by knee osteoarthritis, is a prevalent condition which in most cases can be effectively treated through conservative, non-surgical care involving exercise therapy, education, psychosocial support, and weight loss. However, most people living with chronic knee pain do not receive adequate care, leading to unnecessary use of opiates and surgical procedures.

**Objective:**

Assess the efficacy of a remotely delivered digital care program for chronic knee pain.

**Methods:**

We enrolled 162 participants into a randomized controlled trial between January and March 2017. Participants were recruited from participating employers using questionnaires for self-assessment of their knee pain, and randomized into treatment (n=101) and control (n=61) groups. Participants in the treatment group were enrolled in the Hinge Health digital care program for chronic knee pain. This is a remotely delivered, home-based 12-week intervention that includes sensor-guided exercise therapy, education, cognitive behavioral therapy, weight loss, and psychosocial support through a personal coach and team-based interactions. The control group received three education pieces regarding self-care for chronic knee pain. Both groups had access to treatment-as-usual. The primary outcome was the Knee Injury and Osteoarthritis Outcome Score (KOOS) Pain subscale and KOOS Physical Function Shortform (KOOS-PS). Secondary outcomes were visual analog scales (VAS) for pain and stiffness respectively, surgery intent, and self-reported understanding of the condition and treatment options. Outcome measures were analyzed by intention to treat (excluding 7 control participants who received the digital care program due to administrative error) and per protocol.

**Results:**

In an intent-to-treat analysis the digital care program group had a significantly greater reduction in KOOS Pain compared to the control group at the end of the program (greater reduction of 7.7, 95% CI 3.0 to 12.3, *P*=.002), as well as a significantly greater improvement in physical function (7.2, 95% CI 3.0 to 11.5, *P*=.001). This was also reflected in the secondary outcomes VAS pain (12.3, 95% CI 5.4 to 19.1, *P*<.001) and VAS stiffness (13.4, 95% CI 5.6 to 21.1, *P*=.001). Participants’ self-reported likelihood (from 0% to 100%) of having surgery also reduced more strongly in the digital care program group compared to the control group over the next 1 year (–9.4 percentage points, pp, 95% CI –16.6 to –2.2, *P*=.01), 2 years (–11.3 pp, 95% CI –20.1 to –2.5, *P*=.01), and 5 years (–14.6 pp, 95% CI –23.6 to –5.5, *P*=.002). Interest in surgery (from 0 to 10) also reduced more so in the digital care program compared to control group (–1.0, 95% CI –1.7 to –0.2, *P*=.01). Participants’ understanding of the condition and treatment options (on a scale from 0 to 4) increased more substantially for participants in the digital care program than those in the control group (0.9, 95% CI 0.6 to 1.3, *P*<.001). In an analysis on participants that completed the intervention (per protocol analysis) all primary and secondary outcomes remained significant at greater effect magnitudes compared to intention to treat, with those completing the program showing a 61% (95% CI 48 to 74) reduction in VAS pain compared to 21% (95% CI 5 to 38) in the control group (*P*<.001). Accounting for the cost of administering the program, we estimate net cost savings on surgery alone of US $4340 over 1 year and $7900 over 5 years for those participants completing the digital care program compared to those in the control group receiving treatment-as-usual. In an exploratory subgroup analysis including only participants exhibiting clinical symptoms of osteoarthritis the program proved equally effective.

**Conclusions:**

This trial provides strong evidence that a comprehensive 12-week digital care program for chronic knee pain, including osteoarthritis, yields significantly improved outcomes for pain, physical function, stiffness, surgery risk, and understanding of the condition, compared to a control group.

**Trial Registration:**

International Standard Randomized Controlled Trial Number (ISRCTN) 13307390; http://www.isrctn.com/ISRCTN13307390 (Archived by WebCite at http://www.webcitation.org/6ycwjGL73)

## Introduction

### Background

Chronic knee pain (CKP), often caused by knee osteoarthritis, affects 1 in 4 individuals over the age of 55 [1], and is a major health condition [2] that is becoming increasingly prevalent [3]. The effects of CKP are far-reaching and not limited only to the knee joint. Rather, chronic pain can result in negative effects on general health status including social functioning, energy and vitality, general health perception, limitations due to emotional and physical problems [4], negative effects on quality of life [5], productivity [6], emotional well-being [7], and health care costs [8].

Current recommendations for management of chronic pain suggest that treatments addressing multiple aspects of pain, including physical, psychological, and social, are most effective as compared to a single therapy [9,10]. Recommended components of effective non-pharmacological care for chronic musculoskeletal pain include physical activity, patient education, weight reduction, and self-management and coping strategies [9,11–13]. Thus, an effective treatment algorithm for CKP is a comprehensive program consisting of the main components of recommended conservative care.

Such comprehensive programs for chronic pain - including knee osteoarthritis (OA), one of the most common diagnosis for CKP [11] - have been shown to improve pain and function [15–23] and reduce utilization of total knee arthroplasty (TKA) [12]. However, despite research into comprehensive programs for CKP, utilization of such programs outside of the research arena is rare. For example, it is estimated that 80% of individuals with CKP due to knee OA are not adequately treated with conservative care [13]. This, in turn, leads many patients to undergo costly knee surgeries that could have been otherwise avoided [14]. Thus, there is a significant need to improve access and increase use of a comprehensive treatment program for the large population of individuals affected by CKP.

Digital technology has the potential to effectively provide comprehensive CKP programs. A digital care program (DCP) incorporating multiple components of recommended care could allow for more efficient, effective, and economical treatment by overcoming barriers to behavior change often observed in traditional in-person care, such as travel time, missed work, cost of care, and limited access to healthcare. Furthermore, a DCP incorporating remote sensing would allow for monitoring of patient adherence, a critical barrier limiting long-term effectiveness of treatment programs [15,16]. Only a few studies, however, have examined the use of digital technologies for CKP, investigating web-based platforms for physical activity and exercise [17,18], pain coping training [19], and more comprehensive programs incorporating education and exercise [20-22]. In particular, there are limited studies using a more rigorous randomized controlled design [18,19,22], and the use of digital health in musculoskeletal conditions is regarded as early stage [23].

We have developed a 12-week program for CKP called the Hinge Health DCP [24]. It consists of recommended components of non-pharmacological care for chronic musculoskeletal pain and includes sensor-guided exercise therapy promoting local muscle strengthening and stretching, education, cognitive behavioral therapy, psychosocial support through teams and personal health coaches, weight loss, and activity tracking. We have previously shown that the Hinge Health 12-week DCP improves clinical outcomes of pain, function, and stiffness over a period of 6 months after initiation of the program in a single-arm study of individuals with CKP [24]. The purpose of this study was to assess the short-term effectiveness (12 weeks after initiation) of the Hinge Health DCP in improving knee pain and disability in subjects with CKP, as compared to a control group receiving treatment as usual and knee care education only. We employed a randomized controlled trial with the hypothesis that the DCP would cause a greater improvement in outcome than the control treatment.

## Methods

### Study Design

This study was a two-armed, randomized, controlled, unblinded trial of participants with chronic knee pain. Online applications were invited from employees and their dependents at participating employers spread out over 12 office locations. Participants were recruited through emails and posters distributed through the participants’ employers between January and March 2017. The trial was approved by the Western Institutional Review Board, and participants completed the intervention at home. The trial was performed in compliance with the Helsinki Declaration for research involving human subjects and in line with ICH-GCP guidelines. The trial was preregistered at International Standard Randomized Controlled Trial Number (ISRCTN) 13307390. We followed CONSORT guidelines for reporting this trial.

### Study Population

We assessed the eligibility of all applicants that completed the baseline questionnaire for CKP through their web browser. Participants provided informed consent as part of this questionnaire using a checkbox and digital information sheet. The inclusion criteria were: (1) age over 18, (2) knee pain for at least 1 month in the last 12 months, (3) participating in the collaborating employers’ health plans, and (4) provision of informed consent. The exclusion criteria were (1) a prior diagnosis of rheumatoid arthritis, (2) surgery on the knee less than 3 months ago, and (3) an injury to the knee less than 3 months ago. We did not include knee OA as an inclusion criterion, though we did assess the presence of OA through 6 self-reported clinical criteria, whereby 3 or more positive criteria suggested OA: age over 50 years, stiffness for <30 minutes in the morning, crepitus, bony tenderness, bony enlargement, and no palpable warmth [11]. As there were a limited number of places available on the program, eligible applicants were prioritized for enrollment, with those exhibiting greater pain, disability, and surgery intent prioritized over those showing less. Applicants that were not prioritized were placed on the waitlist (n=73). Participants were not paid for their time, other than an incentive offered to complete the outcome questionnaire for those that did not complete it within 4 days of first invitation.

### Randomization

Eligible applicants were randomized into the trial twice weekly during the signup period. Batches of selected participants were then randomized into treatment and control using a 60:40 (treatment: control) ratio (n=115) or using an 80:20 ratio (n=47). The 80:20 ratio represents a deviation from the study protocol due to an administrative error and was only used for a restricted time. The effective allocation ratio was therefore 62:38 (treatment: control). When a batch of applicants was randomized, an algorithm shuffled the batch and selected the first 60% (or 80%) to enter the treatment, and the remaining 40% (or 20%) to enter control. As such, the person(s) reviewing the applicants had no way of knowing whether any given applicant would end up in the treatment or control group (concealed allocation). After randomization, participants in the treatment group received an email inviting them to complete their profile and receive their kit to participate in the DCP, whereas those in the control group received an email with three education articles to help them care for their knee. Due to the nature of the study, neither the study staff nor the participants were blinded to group allocation.

### Study Intervention

The treatment group received the Hinge Health 12-week DCP for CKP. The contents of this program have been described previously [24]. In short, participants received a tablet computer with the Hinge Health application installed, and two custom Bluetooth sensors with straps to be used on the upper and lower leg during the in-app exercise therapy. Participants were assigned a personal coach that provided support and accountability throughout the program and were placed in a team to provide peer support through a discussion feed within the app. Participation was completed entirely remotely through the app, at times and places chosen by the participant. Reminders were provided by text message and email if the participant was not engaging at the recommended intensity with the program. On a weekly basis, participants in the DCP were set the goal of completing 3 sessions of sensor-guided exercise therapy, reading one to two education articles, logging their symptoms at least twice, performing cognitive behavioral therapy (CBT; subset of weeks only), working at weight loss (if overweight), and tracking at least three 30-minute sessions of aerobic activities. Details of each of these components of the DCP are described elsewhere [24]. Each participant also maintained access to treatment as usual (TAU).

The control group received three pieces of education, presented digitally, that is also part of the Hinge Health DCP. These articles discussed the importance of self-care, how to deal with setbacks in knee pain, and how to manage communication and relationships when living with CKP. The control group maintained access to TAU and were informed that they would be reconsidered for the program when new places became available following the 12-week study.

The application was developed, owned, and sponsored by Hinge Health, Inc. The 12-week program received extensive testing over a 2-year period prior to starting the trial. All participants received the same version of the program, and there were no major application updates during the course of the trial.

### Study Outcomes

#### Primary Outcomes

The preregistered primary outcomes were the Knee Injury and Osteoarthritis Outcome Score (KOOS) Pain subscale [25], and the KOOS Physical Function Shortform (KOOS-PS, referred to as “KOOS short version” in preregistration) scale [26]. Both scales span from 0 (no pain or impaired function, respectively) to 100 (extreme pain or impaired function, respectively), and were assessed at baseline and at the end of the 12-week DCP in the intervention and control groups. Both surveys are composite measures which may confound multiple conditions, however the digital nature of the program precluded individual clinical evaluation. Additionally, those in the treatment group were asked to complete both questionnaires at weeks 4 and 8 as part of the DCP. To conclude a positive effect of treatment we required a significant effect on *both* primary outcomes, though we note this was not specified in the preregistration.

#### Secondary Outcomes

We describe multiple preregistered secondary outcomes. Firstly, a visual analogue scale (VAS) for the question “Over the past 24 hours, how bad was your knee pain?” from 0 (“none”) to 100 (“worst imaginable”). Secondly, a VAS for the question “Over the past 24 hours, how bad was your knee stiffness?” from 0 (“none”) to 100 (“worst imaginable”). Thirdly, we assessed surgery intent using multiple questions: “What do you think are the chances you'll have knee surgery in the next year, in %?” as well as the same question for 2 and 5 years into the future. We also asked “On a scale of 0 to 10 how interested are you in knee surgery?” with labels “not at all” at 0, and “definitely going to get surgery” at 10. Lastly, we asked “Thinking about your symptoms, how well do you feel you understand your condition and your treatment options?” with answers “Not at all”, “Slightly”, “Moderately”, “Very well”, “Completely”, coded from 0 to 4. All data were assessed at baseline and at the end of the 12-week DCP in both the intervention and control groups. Additionally, those in the treatment group were asked to complete these questions at various points during the DCP: the VAS twice each week, and the questions related to surgery and understanding of their condition at week 6.

### Sample Size

The minimal clinically important difference for KOOS is considered to be approximately 10 points on the 100-point scale, and a standard deviation of 15 is recommended for power calculations [27]. Although we did not use the full KOOS scale, we assumed its derivate questionnaires would have similar properties and used a standard deviation of 15 to perform power calculations. The number of participants needed in each group to detect a 10-point difference given a Type I error rate of 0.05 and power of 0.8 is 36. Given our unequal allocation ratio, this would need to be at least 54 in the treatment group and 36 in the control group for a total of at least 90 participants in the trial. As we had two primary endpoints albeit not independent of one another, we significantly over-recruited participants into the trial. We opted for an unequal allocation ratio to ensure we would be able to enter a certain minimum number of people into the treatment arm, a criterion mandated by the commercial nature of the deployments.

### Statistical Analysis

Our primary analysis was conducted using a modified intent-to-treat approach. This analysis included all participants that were randomized, including those in the treatment group that never started the DCP. However, we excluded 7 participants in the control group that were enrolled in the DCP due to an administrative error (including these participants does not materially affect the statistical significance of the results). We describe baseline characteristics for the treatment and control groups using frequencies, means, and standard deviation. For those participants in the treatment group that performed at least one session of exercise therapy and those that completed the week 12 outcome questionnaire respectively, we also provide descriptive statistics of their engagement with the DCP. The analysis of primary and secondary outcomes was performed using a linear mixed model using the Linear Mixed-Effects Model (“lme4”) package in R [28] with within-subject factor “time point” (baseline or outcome) and between-subject factor “group” (treatment or control) and their interaction. We modeled a separate baseline for each participant, effectively examining the change scores only (in lme4 this was performed as “score ~ timepoint*group + (1|participant)”, where (1|participant) models an intercept for each participant separately). We assessed normality of the residuals based on quantile-quantile (QQ)-plots. If we did not have outcome data for a participant, we used last observation carried forward (LOCF). For those in the control group, this meant their baseline was carried forward; for those in the treatment group this meant either their baseline was carried forward, or data collected during the course of the DCP. We also analyzed all primary and secondary outcomes with baseline carried forward (BOCF) also for the treatment group (rather than LOCF). We also omitted LOCF and instead allowed the mixed-effects model to account for the missing data, which yielded an identical pattern of results as using LOCF and BOCF. We also report the primary and secondary outcomes following a per-protocol analysis to assess the effect of the program on those that complete it. Lastly, we performed an exploratory subgroup analysis using the same primary and secondary outcomes on participants that met the criteria for knee OA as defined by having at least 3 out of 6 clinical criteria: age >50 years, stiffness in the morning <30 min, crepitus, bony tenderness, bony enlargement, and no palpable warmth [11].

### Surgery Cost Savings

We report the expected savings on surgery costs based on participants’ self-assessment of their likelihood to have surgery. The calculation estimates the cost of knee surgery at US $40,000 [29]. For example, a 10-percentage point reduction in self-reported 1-year surgery likelihood would translate into a cost saving of US $4000 in the first year, minus the costs of the program per participant. The net cost saving is not considered a primary or secondary outcome of the clinical trial and is only calculated for those participants completing the trial (per protocol).

## Results

### Study Population

**Figure 1:**
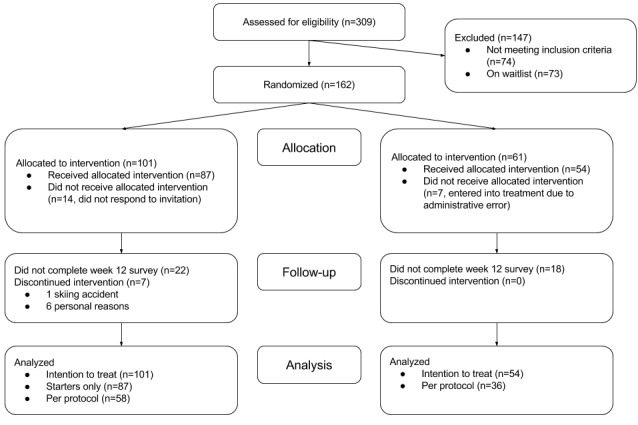
Participant flow diagram.

A total of 309 people completed the screening for CKP in January or February 2017, of which 162 entered the trial and were randomized (Figure 1). Of those 162 individuals, 62% entered the treatment arm (101/162) and 38% entered the control arm (61/162). Seven participants in the control arm were given the DCP due to administrative error and have all been excluded from all following results. The errors afflicted this set of participants completely at random (ie, not as a function of symptoms, demographics, or otherwise) and their exclusion, therefore, does not bias the findings; in contrast, including these participants would have led to an underestimation of the true effect of treatment. The baseline demographics were comparable between groups (Table 1), with the average participant 46 years of age and overweight. At baseline, all but 1 participant believed the DCP would help them delay surgery, and 87% (135/155) believed the DCP could help them avoid surgery altogether. A substantial minority (41%) had already undergone knee surgery in the past, though none were actively rehabilitating. The only difference in demographics between both groups was the gender balance; there were more women in the treatment compared to control group (43% versus 26% respectively).

**Table 1:** Demographics of the control and treatment groups. The term SD refers to standard deviation.

Characteristic	Treatment	Control	All
Number of participants	101	54	155
Age in years, mean (SD)	46 (12)	47 (12)	46 (12)
Body Mass Index (kg/m2), mean (SD)	27 (5)	28 (4)	27 (5)
Female, n (%)	43 (43)	14 (26)	57 (37)
Physical Training-like exercise at screening^a^, n (%)	33 (33)	17 (31)	50 (32)
Fear avoidance^b^, n (%)	6 (6)	6 (11)	12 (8)
Godin activity score^c^, mean (SD)	34 (23)	39 (25)	36 (24)
Hours sedentary per day, mean (SD)	6 (3)	6 (3)	6 (3)
Think Digital Care Program can delay surgery, n (%)	100 (99)	54 (100)	154 (99)
Think Digital Care Program can avoid surgery, n (%)	87 (86)	48 (89)	135 (87)
Taking antidepressants, n (%)	2 (2)	0 (0)	2 (1)
Taking opioids, n (%)	5 (5)	3 (6)	8 (5)
Self-efficacy^d^, mean (SD)	11 (3)	10 (3)	11 (3)
Surgery on the knee in the past, n (%)	45 (45)	19 (35)	64 (41)
Knee osteoarthritis^e^, n (%)	75 (74)	44 (81)	119 (77)

^a^Positive answer to the question “Do you currently do any physical therapy-style exercises?”

^b^Positive answer to the question “It's really not safe for a person with a condition like mine to be physically active.”

^c^Composite score; 24 indicates “active”, 14-23 indicates “Moderately active”, and <14 indicates “Insufficiently active/sedentary” [30].

^d^Health self-efficacy assessment, scores from 0 (no self-efficacy) to 15 (high self-efficacy) [31].

^e^Defined as satisfying at least 3 out of 6 clinical criteria for osteoarthritis [11].

### Participant Flow

Participants in the treatment group were lost at three stages. First, of the 101 participants randomized to treatment, 14 did not respond to our invitation to take part in the DCP or subsequent follow-up communications. Second, 7 participants actively withdrew during the course of the DCP, due to injury or for personal reasons (eg, time constraints or stress at work, Figure 1). Third, 22 participants did not complete the week 12 online outcomes survey.

In the control group, 7 participants were placed in the DCP due to an administrative error. A further 18 participants did not complete the week 12 outcomes survey.

### Engagement

For participants in the treatment group, we tracked completion of each component of the DCP. Participants that started the DCP (n=87), defined as performing at least one sensor-guided workout, performed an average of 33 in-app workouts, or an average of 2.5 workouts per week from week 0 (introduction to the program) to 12. Users that completed the outcome questionnaires at 12 weeks (n=59) performed 43 sensor-guided workouts (3.3 workouts per week), compared to the 3 times per week that is recommended in the DCP. Average weekly engagement with the DCP was 76% for those that started the program, and 95% for those that completed it. Participants that completed the 12-week follow-up read approximately 10 education articles, completed 2 Cognitive Behavioral Therapy (CBT) sessions, posted on the feed 8 times, and contacted their coach over text message or in-app message about 7 times.

### Outcomes

#### Primary Outcomes

Both primary outcomes improved significantly more in the treatment group compared to the control group (Table 3). We observed a statistically significant mean group difference for KOOS Pain whereby the treatment group improved by 7.7 (95% CI 3.0 to 12.3) points more than the control group. Similarly, the treatment group improved by 7.2 (95% CI 3.0 to 11.5) points more than the control group on the KOOS-PS scale.

#### Secondary Outcomes

Each of the secondary outcomes also showed the DCP to be superior to control (Table 3). The VAS pain and stiffness scores, although improved in the control as well as the treatment group, improved by 12 (95% CI 5.4 to 19.1) points more for those in the DCP than in control. The self-reported likelihood of surgery in the next 1, 2, and 5 years, as well as interest in having surgery, all decreased more for those in the DCP than those in control over the 12-week period. Lastly, participants on the DCP improved their understanding of their condition and treatment options more than those in the control group. All primary and secondary outcomes remained statistically significant in an analysis using baseline carried forward for the treatment group rather than last observation carried forward.

### Per Protocol Analysis

We also provide results for only those participants that received their allocated intervention and completed the outcome questionnaires (n=58 for treatment, n=36 for control) in Table 4. The per protocol results are fully consistent with the intent-to-treat analysis.

Based on the reductions in surgery likelihood we also calculated the net savings per participant of the program after accounting for the costs of delivering the program. The 1-year net saving of the digital care program is US $4340 (13.1% * US $40,000, corrected for the cost of the digital care program), the 2-year savings are US $4660, and the 5-year savings are US $7900.

### Subgroup Analysis: Knee Osteoarthritis

An intent-to-treat analysis on participants with knee osteoarthritis (75/101 in the treatment group; 44/54 in the control group) showed results consistent with the original intent-to-treat analysis presented in Table 3 in terms of the magnitude of the group difference and statistical significance.

**Table 2:** Engagement indicators for each of the aspects of the Digital Care Program. “Starters” indicates participants performed at least one sensor-guided workout. “Finishers” indicates participants that completed the outcomes questionnaires at week 12 follow-up.

Indicator	All Starters (n=87)	All Finishers (n=59)
Number of workouts, mean (SD)	33.1 (24)	42.9 (17.3)
Users engaging with the program per week, n (%)	66.3 (76.2)	55.9 (94.8)
Users active with sensor-guided exercise in weeks 1-4, n (%)	78 (89.7)	58 (98.3)
Users active with sensor-guided exercise in weeks 5-8, n (%)	69 (79.3)	56 (94.9)
Users active with sensor-guided exercise in weeks 9-12, n (%)	60 (69)	55 (93.2)
Offline activities logged in hours, mean (SD)	9.6 (9.1)	13.2 (8.8)
Education articles read, mean (SD)	7.3 (4.5)	9.6 (3.1)
Cognitive Behavioral Therapy session completed, mean (SD)	1.4 (1.2)	1.8 (1.1)
Team posts and comments, mean (SD)	6.1 (7.2)	8.4 (7.6)
In-app messages sent to coach, mean (SD)	5.9 (5.6)	6.6 (5.7)

**Table 3:** Primary and secondary outcomes for the intent-to-treat analysis of treatment and control groups. The mean group difference as well as the *P* value for the interaction are derived from the linear mixed effects model. Each of the primary and secondary outcomes favors the treatment over the control group. *P* values uncorrected for multiple tests. KOOS: Knee injury and Osteoarthritis Outcome Score; PS: Physical Function Shortform; VAS: visual analogue scale.

Outcome		Treatment at baseline, mean (SD)	Treatment at outcome, mean (SD)	Control at baseline, mean (SD)	Control at outcome, mean (SD)	Group difference, mean (95% CI)	Interaction, *P* value
**Primary Outcomes**						
	KOOS Pain (0-100)	41.0 (14.1)	30.3 (17.1)	41.4 (16.5)	38.4 (17.2)	–7.7 (–12.3 to –3)	.002
	KOOS-PS (0-100)	53.8 (12.3)	44.6 (16.7)	54.5 (15.7)	52.5 (16.2)	–7.2 (–11.5 to –3)	.001
**Secondary Outcomes**						
	VAS Pain score (0-100)	45.2 (21.4)	26.6 (22)	44.7 (20.3)	38.3 (22.2)	–12.3 (–19.1 to –5.4)	.001
	VAS Stiffness score (0-100)	42.6 (23.4)	25.1 (22.3)	47.4 (21.9)	43.2 (21.6)	–13.4 (–21.1to –5.6)	.001
	Surgery chance next year, %	24.5 (26.9)	14.7 (25)	24.3 (26.2)	23.9 (29.1)	–9.4 (–16.6 to –2.2)	.01
	Surgery chance next two years, %	32.1 (31)	19.1 (26.9)	31.7 (27.9)	30 (28.9)	–11.3 (–20.1 to –2.5)	.01
	Surgery chance next five years, %	47.8 (35)	27.5 (32.9)	49.8 (32.7)	44.1 (33.6)	–14.6 (–23.6 to –5.5)	.002
	Surgery interest (0-10)	3.03 (3.41)	1.92 (2.93)	3.02 (3.32)	2.89 (3.21)	–1.0 (–1.7 to –0.2)	.01
	Understanding of condition and treatment options (0-4)	1.92 (1.01)	2.68 (0.937)	1.94 (1.04)	1.76 (1.03)	0.9 (0.6 to 1.3)	<.001

**Table 4:** Per protocol results. All participants that completed their assigned treatment and completed the week 12 outcome questionnaire are included. KOOS: Knee Injury and Osteoarthritis Outcome Score; PS: Physical Function Shortform; VAS: visual analogue scale.

Outcome		Treatment at baseline, mean (SD)	Treatment at outcome, mean (SD)	Control at baseline, mean (SD)	Control at outcome, mean (SD)	Group difference, mean (95% CI)	Interaction, *P* value
**Primary outcomes**						
	KOOS Pain (0-100)	39.6 (14.5)	21.8 (13.4)	39.2 (14.7)	34 (12.9)	–12.6 (–18.7 to –6.5)	<.001
	KOOS-PS (0-100)	52.9 (12.6)	37.4 (16.1)	51.8 (16.4)	48.4 (15.9)	–12.1 (–17.7 to –6.6)	<.001
**Secondary outcomes**						
	VAS Pain score (0-100)	44.1 (21.5)	17.2 (16.2)	45.5 (19.6)	35.8 (21.8)	–17.3 (–26.3 to –8.3)	<.001
	VAS Stiffness score (0-100)	42.4 (24.3)	15.9 (17.3)	47.4 (22.1)	40.8 (20.9)	–19.9 (-30.4 to –9.4)	<.001
	Surgery chance next year, %	21.6 (24.9)	7.59 (18.5)	20.8 (21.9)	20 (26.3)	–13.1 (–24.1 to –2.2)	.02
	Surgery chance next two years, %	28.1 (29.1)	12.1 (21.5)	27.4 (25)	25.3 (26.9)	–13.9 (–26.6 to –1.3)	.03
	Surgery chance next five years, %	48.7 (33.9)	18.2 (26.5)	48.6 (29.9)	40.1 (30.9)	–22 (–35 to –9.1)	.001
	Surgery interest (0-10)	2.93 (3.28)	1.31 (2.39)	3 (3.3)	2.78 (3.14)	–1.4 (–2.5 to –0.3)	.01
	Understanding of condition and treatment options (0-4)	1.88 (1.03)	3.09 (0.657)	1.83 (1.06)	1.56 (0.998)	1.5 (1.1 to 1.9)	<.001

## Discussion

### Principal Findings

While CKP is a prevalent cause of disability worldwide [1,2], comprehensive conservative programs for the condition are lacking. The Hinge Health DCP has been designed to address this lack of chronic pain programs and incorporates components of best-practice conservative care for CKP in a digital format that provides flexibility to the user. The results of this randomized controlled trial demonstrated large improvements in knee pain, physical function, and stiffness in individuals with CKP on the Hinge Health DCP that were significantly greater than a control group receiving knee care education and treatment as usual over a period of 12 weeks after program initiation. Participant surgery interest also significantly decreased and understanding of their condition increased in the treatment group as compared to the control group. The positive results of this study demonstrate the potential of the Hinge Health DCP as a treatment for a large number of individuals affected by CKP that otherwise would be at risk for surgery.

Analysis of primary study outcomes demonstrated large improvements in both KOOS pain and function scales in the treatment group. Similarly, significantly greater improvements in physical function (KOOS-PS) scores were observed in the treatment group as compared to the control group. When considering individuals who started on and completed the study as per protocol, the improvements observed in KOOS Pain and function scores were 45% in the treatment group as compared to 13% in control, and 29% in treatment as compared to 7% in control, respectively. The improvements observed in the treatment group exceeded recommended minimal clinically important changes for KOOS [27], while the small improvements in the control group did not. The group difference in KOOS did not quite reach the minimal clinically important difference due to the relatively large drop-off which ‘diluted’ the improvements of those that completed the program. Nonetheless, the clinically significant improvements demonstrated at the end of the 12-week program for not only the primary outcome measures but also secondary metrics provide strong evidence for the benefits of the Hinge Health DCP for individuals affected by CKP.

Similar large improvements that were significantly greater in the treatment group than in control were noted in secondary outcomes of VAS pain and stiffness scales. At the end of the 12-week program, per protocol participants in the treatment group had 61% and 63% improvements in VAS pain and stiffness, as compared to 21% and 14%, respectively, in the control group. Subjects’ perception of surgery interest and surgery requirements also changed favorably at the end of the 12-week Hinge Health DCP, with a 63% reduction in the belief that they would require surgery within the next 5 years in the per protocol treatment group as compared to a reduction of 17% in the control group. It is also important to note that the Hinge Health DCP was safe for participants, as there were no reported adverse events during the 12-week program.

The results of this study demonstrated comparable or greater improvements in pain, physical function, and stiffness as compared to other treatment programs for CKP. Bossen et al [18] demonstrated improvements of 15% in physical function and 35% in pain at 3 months after initiation of a 9-week web-based behavior graded physical activity intervention in patients with knee and/or hip OA. Hughes et al [32] found improvements in Western Ontario and McMaster Universities Osteoarthritis Index (WOMAC) pain, stiffness, and function scores of 23%, 17%, and 23%, respectively, at the end of an 8-week exercise and behavior-change program for OA. Similarly, Hurley et al [33] demonstrated per protocol improvements in WOMAC pain and function of 31% and 26%, respectively, at the end of a 6-week rehabilitation program combining self-management and exercise for CKP. While the current study did not investigative the longer-term effect of the Hinge Health DCP past the end of the 12-week program, data from prior studies of treatment programs of similar duration (6-12 weeks) [18,22,32-34] showed improvement in outcomes can be maintained as long as 30 months after program completion [33]. Thus, it is likely that the results of the Hinge Health DCP would be maintained after the 12-week program.

The improvements observed as a result of the Hinge Health DCP have the potential to translate into substantial economic savings, however, in lieu of long-term data are based on participant self-report. Based on results for participants’ self-reported likelihood of having surgery, the potential savings per completing participant due to surgery avoidance alone equate to US $4340 net cost savings on surgery over the first year in individuals using the Hinge Health DCP as compared to treatment as usual. Other integrated rehabilitation programs for CKP have also demonstrated lower healthcare costs as compared to usual care [33]. Thus, while the long-term effect of the Hinge Health DCP may in part be dependent on continued adherence to the program [35], it is anticipated that the behavioral, educational, and psychosocial components of the program have the potential for long-term clinical and economic effects [33].

When interpreting the results of this study, its strengths and limitations should be considered. Strengths of this study include the randomized controlled study design, and that the study was designed, conducted, and analyzed according to a pre-specified protocol. Further, the digital format of the program provides flexibility to individuals to participate at times and places convenient to them. While the results of this study demonstrate significantly greater improvements in primary and secondary outcomes with the Hinge Health DCP as compared to control, this study did not investigate the long-term effect of the Hinge Health DCP past the end of the 12-week program. Thus, further work is needed to evaluate the long-term impact of the Hinge Health DCP as compared to control. Our prior work suggests the potential for long-term effects as it demonstrated improved patient-reported outcomes in a single-arm study 6 months after program initiation [24]. We are in the process of collecting multi-year data and these results will be published in due course. A second point to note is that preliminary analyses not shown here suggest that BMI, gender, and surgery risk all affect the risk of dropping out. We plan to investigate risk factors for failure to adhere to the DCP in an upcoming study. Finally, around 20% of participants had less than 3 months of pain over the past 12 months, which does not strictly meet a definition of chronic pain. In a larger cohort, the efficacy of the program on long-term versus intermittent knee pain should be examined.

The study enrolled a representative population with CKP problems. While CKP is a hallmark symptom of knee OA [11], a diagnosis of knee OA was not required for inclusion in this study. Analysis of participant baseline data demonstrated that 74% of individuals in the treatment arm and 80% of individuals in the control arm had knee OA as defined by clinical diagnosis for knee OA derived from the American College of Rheumatology criteria for OA of the knee [11]. A sub-group analysis of these participants confirmed the successful outcome of the Hinge Health DCP in the primary and secondary outcomes over the 12-week period as compared to control, demonstrating the applicability of the program to highly prevalent knee OA.

The comprehensive nature of the Hinge Health DCP addresses multiple components of recommended management for CKP [9,10]. However, it is therefore unknown if all components of the program (sensor-guided exercise therapy, education, cognitive behavioral therapy, weight loss, and psychosocial support) are necessary to attain the reported results. Similar to other studies investigating interventions for CKP and knee OA, [18,22,32,34,36] due to the nature of this study, participants could not be blinded as to the intervention, and thus we cannot rule out the possibility of an attention effect. The attrition rate for week 12 patient-reported outcomes was in line with other studies for CKP [18]. Further, as the rate was similar in both control and treatment it is not anticipated to have impacted the findings of the study.

### Conclusion

Individuals with CKP who used the Hinge Health DCP for 12 weeks experienced significantly greater improvement in self-reported clinical outcome measures of pain, physical function, stiffness, as well as surgery intent and understanding of their condition, as compared to a control group receiving knee education articles and treatment as usual. Given the observed benefits, the Hinge Health DCP may be an effective comprehensive treatment program for individuals with CKP.

## Appendix

Multimedia Appendix 1 CONSORT‐EHEALTH checklist (V 1.6.1).

## Abbreviations

BMI: body-mass index
CBT: cognitive behavioral therapy
CKP: chronic knee pain
CONSORT: Consolidated Standards of Reporting Trials
DCP: digital care program
KOOS: Knee injury and Osteoarthritis Outcome Score
OA: osteoarthritis
PS: Physical Function Shortform
VAS: visual analogue scale
